# De novo AML spontaneously achieved PR after COVID-19 infection, and CR after reduced dose of azacytidine combined with venetoclax: A case report and literature review

**DOI:** 10.1097/MD.0000000000042039

**Published:** 2025-04-25

**Authors:** Yao Qi, Jing-Yi Li, Jia Wang, Juan Mu, Qi Deng, Rui Cui

**Affiliations:** a Department of Hematology, Tianjin First Central Hospital, School of Medicine, Nankai University, Tianjin, China; b College of Tianjin Medical University, Tianjin, China.

**Keywords:** acute myeloid leukemia, antitumor effect, azacitidine, COVID-19, venetoclax

## Abstract

**Rationale:**

Coronavirus disease (COVID-19) infection increases the mortality of patients with hematological malignancies. The optimal treatment for de novo acute myeloid leukemia (AML) patients with severe pneumonia caused by COVID-19 is not clear.

**Patient concerns:**

A 59-year-old woman was admitted to our department with fever, cough dyspnea, and thrombocytopenia for 1 week.

**Diagnoses:**

The patient was diagnosed with AML associated with a *TP53* mutation and complex chromosomal abnormalities by bone marrow examination. In addition, she had severe COVID-19 pneumonia when her AML was diagnosed.

**Interventions:**

We delayed leukemia therapy to adequately treat her severe COVID-19 pneumonia. In the therapy for COVID-19 pneumonia, the patient presented with high levels of tumor necrosis factor-α and interleukin 6. Surprisingly, after being treated for severe COVID-19 pneumonia, she obtained partial remission in the absence of leukemia therapy. When the severe COVID-19 pneumonia was under control, the patient achieved complete remission after she received a reduced dose of azacytidine combined with venetoclax for only 1 cycle.

**Outcomes:**

After a standard dose of azacytidine combined with venetoclax for 2 cycles, the patient achieved a deep molecular remission. The results of next-generation sequencing analysis indicated that the *TP53* mutation turned negative.

**Lessons:**

This case suggests that azacytidine combined with venetoclax could be a safe and valid option compared with intensive chemotherapy in newly diagnosed AML patients with severe COVID-19 pneumonia. Whether the increased cytokine levels could indicate that COVID-19 infection might have an anti-tumor effect on AML patients remains to be further observed.

## 1. Introduction

The therapy of newly diagnosed acute myeloid leukemia (AML) patients during the period of coronavirus disease (COVID-19) could be particularly challenging. AML patients have myelosuppression and immunosuppression after chemotherapy, making them to be susceptible to infection with COVID-19 and may have serious complications.^[1,2]^ At the time of diagnosis or during the treatment of AML, patients might experience COVID-19 infection which has a significant impact on the likelihood of receiving the best standard chemotherapy. Torron et al recommended initiation of chemotherapy for AML patients following the resolution of COVID-19 symptoms and achievement of a negative polymerase chain reaction (PCR) test.^[3]^ However, the direct influence of COVID-19 infection on newly diagnosed AML is still unclear now. Due to there are no unified guidelines to guide the optimal therapy for de novo AML patients with serious COVID-19 infection, here we report on a typical such patient. She spontaneously achieved PR after serious COVID-19 infection, and CR after reduced dose of azacytidine combined with venetoclax.

## 2. Case presentation

**Figure 1.**
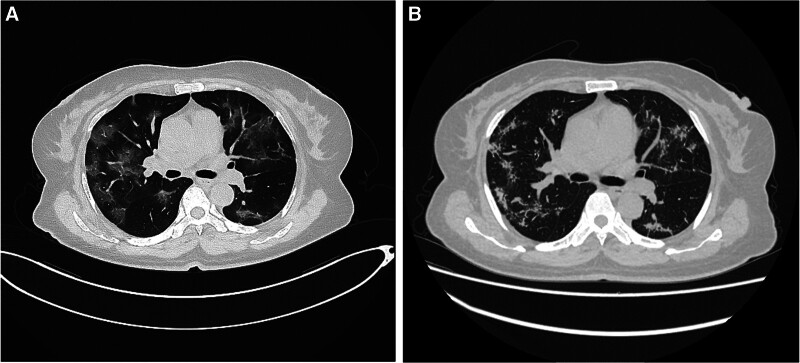
(A) The initial chest CT scan displays patchy infiltrates with extensive patchy areas of ground glass opacification, typical of COVID-19 pneumonia. (B) After 6 weeks from the diagnosis, the Chest CT shows extensive resolution of lung infiltrates, as well as areas of consolidation.

A 59-year-old woman was presented to our department on December 21, 2022 with 1 week of fever, cough dyspnea, and thrombocytopenia. She had no medical history. Her complete blood count was drawn, white blood cell count of 6.5 × 10^9^/L with a monocytic predominance (45%), neutrophil count of 0.2 × 10^9^/L, platelet count of 26 × 10^9^/L, and hemoglobin of 100 g/L. Real-time quantitative PCR test for COVID-19 was positive in this patient. Consistent with COVID-19 pneumonia, her chest computed tomography (CT) showed multiple bilateral patchy alveolar opacities (Fig. 1A). Clinically, the clinical presentation is severe infection, hypoxemia (92 mm Hg), but mechanical ventilation is not required.

**Figure 2.**
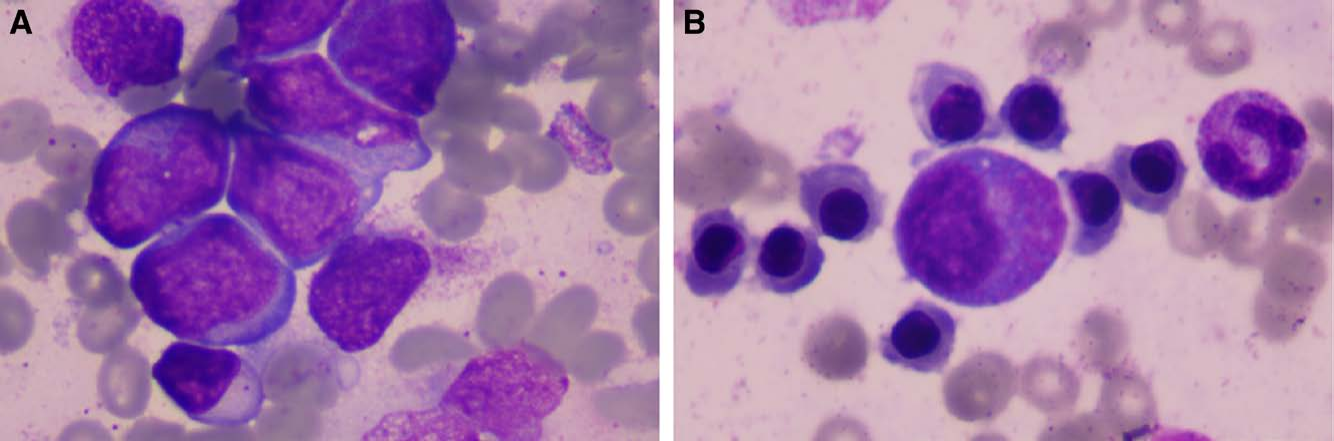
(A) Morphological analysis of the bone marrow smear at diagnosis showed infiltration by myelomonocytic blasts. (B) After 1 cycle of AZA/VEN treatment, bone marrow smear showed morphological complete remission.

Bone marrow (BM) morphology showed 68% blasts with features of abnormal mono monoblasts (Fig. 2A). A flow cytometry (FCM) analysis of BM identified a large population of blasts expressing MPO, CD13, CD33, CD34, CD117, CD38, and CD123. Cytogenetic revealed complex chromosomal abnormalities (46–50, XX, +4, +6, +8, +21/46, XX). Next-generation sequencing (NGS) analysis identified *TP53* (29%) and *NPM1* (25%) mutation. Therefore, according to ELN 2017 recommendations, the disease is classified as a high risk AML.^[4]^

Because of the patient’s combination of severe infection and hypoxemia, leukemia therapy was postponed in order to receive adequate COVID-19 pneumonia supportive treatment. Then, she was started with therapy of meropenem 1000 mg/dose every 8 hour, molnupiravir 0.8 g/dose every 12 hour, and dexamethasone 40 mg/dose every day to treat COVID-19 pneumonia. We observed the cytokine changes in this patient during her COVID-19 therapy. There was an significantly increase in levels of interleukin 6 (IL-6) and tumor necrosis factor-α (TNF-α) levels after COVID-19 infection, and the cytokine levels decreased as the infection was contained (Table 1). Fourteen days later, the symptoms of COVID-19 pneumonia were relieved with negative for COVID-19.

**Table 1:** The changes of proinflammatory cytokines after COVID-19 infection.

	Day 1	Day 5	Day 10	Reference range (pg/mL)
IL-2	6.2	8.8	1.5	0–10.7
IFN-ɤ	24.4	6.4	1.7	0–19.3
IL-10	43.7	13.1	7.8	0–8.2
IL-17	19.1	12.5	4.8	0–20.9
IL-6	432.9	156.4	23.2	0–18.9
IL-4	4.2	3.45	2.9	0–11.2
TNF-α	139	64.4	12.5	0–8.4
IL-1β	33.4	16.5	2.8	0–12.6
IFN-α	23.1	5.1	3.6	0–8.6
IL-12P70	3.2	2.6	0.5	0–3.4
IL-8	205.2	56.4	34.2	0–21.4
IL-5	4.3	7.7	0.2	0–3.5

The patient presented with high levels of IL-6 (range 0–18.9 pg/mL), TNF-α (range 0–8.4 pg/mL).

After 2 weeks of anti-infective therapy, complete blood count was drawn, a total white blood cell count of 3.5 × 10^9^/L, neutrophil count of 0.7 × 10^9^/L, platelet count of 55 × 10^9^/L, and hemoglobin of 84 g/L. The BM assessment showed that the blasts had been decreased to 13%. Surprisingly, the patient achieved partial remission (PR) without receiving chemotherapy to leukemia.

Because of her recent severe of COVID-19 pneumonia, standard intensive induction therapy for acute leukemia was deemed unacceptable. Therefore, she was received azacitidine (AZA, 75 mg/m^2^/dose every day on day 1–7) in combination with venetoclax (VEN 100/dose every day on day 1–7) as induction therapy after discontinuing all anti-infective therapy. The patient was well tolerated during the therapy of AZA combination with VEN, without infectious or hemorrhagic events. Fifteen days after induction therapy, we observed complete recovery of neutrophil and platelet counts. At the end of the induction therapy of AZA combination with VEN, morphological complete remission (CR) was achieved, minimal residual disease (MRD) monitoring by FCM was negative (Fig. 2B). A new chest CT scan was repeated on February 2, 2023 showed extensive absorption of lung infiltrates compared with the previous CT scan (Fig. 1B). Then, the patient received 2 cycles of consolidation therapy of AZA (75 mg/m^2^/dose every day on day 1–28) in combination with VEN (400 mg/dose every day on day 1–28) after her induction therapy. Then, BM morphology indicated that the patient remained in CR. She remained negative MRD by FCM, normal karyotype by chromosomal evaluation, and negative *TP53* mutation by NGS analysis. Next, the patient is prepared to undergo haploid hematopoietic stem cell transplantation.

## 3. Discussion and conclusions

In the COVID-19 pandemic, recent evidence emerged that active hematological malignancies patients with an ascertained COVID-19 diagnosis have more severe outcomes and a higher fatality rate.^[5]^ Data linking the impact of COVID-19 on treatment of AML after COVID-19 remain difficult to evaluate. Complicating factors including age, comorbidities, and COVID-19 pneumonia severity determined the regimens whether to reduce, postpone or continue treatment. ASH and ECIL 9 suggested that if COVID-19 is positive, the induction therapy should not be started until the PCR turned negative.^[6]^ Recently, a clinical study surveyed 388 AML patients, of whom 39% delayed chemotherapy and 61% discontinued therapy.^[7]^ Treatment delayed has a protective effect on the clinical course of COVID-19, while the interruption of treatment is related to a worse outcome in a multivariate analysis.^[7]^ The results of a real-world research about patients with newly diagnosed AML, revealed that no significant difference in the overall survival and CR rate after delaying induction treatment up to 15 days.^[8]^

Base on the proposal of the ESBMT and ASH, AML patients in good status should receive intensive chemotherapy.^[9,10]^ Intensive chemotherapy may lead to prolonged granulocytopenia (often > 3 weeks) and increased severe infection rate.^[11]^ However, to avoid prolonged cytopenia, and to reduce the risk of COVID-19 complications, low-dose therapy with AZA plus VEN could be taken as an alternative option, especially in those patients with poor performance condition.^[12,13]^ An observational study of 139 patients with AML/MDS, the impact of chemotherapy regimens on the gravity and mortality of patients with COVID-19 infection was assessed. The researchers found that mortality was significantly decreased in patients with AML/MDS who received hypomethylating agents (HMA).^[14]^ Compared to HMA alone, adding VEN to HMA significantly enhanced the CR rate and improved the survival in elderly patients with newly diagnosed AML.^[15]^ In this article, we describe a patient with a new diagnosis of AML who had diagnosed with severe COVID-19 pneumonia, achieved CR with 1 cycle of very low-dose VEN combined with AZA. Then after 2 standard cycles of AZA combined with VEN, *TP53* mutation became undetectable, and the deep molecular remission was achieved in this patient. The treatment was well tolerated and verified the applicability of this reduced intensity regimen as induction to AML patients with concomitant COVID-19 pneumonia. Although further research is needed in this setting, AZA/VEN can be regarded as a treatment option for patients with de novo AML and severe COVID-19, where a prolonged period of chemotherapy-induced pancytopenia could adversely affect prognosis. Moreover, the improved quality of clinical response to AZA/VEN pushed us to reflect on the possible effect of the AZA and VEN combination at molecular level.

Of interest, this patient showed partial spontaneous remission (SR) before induction therapy of AML. SR is a rare event in a newly diagnosed AML without chemotherapy. SR after COVID-19 infection in patients with hematological malignancies have been reported in the literature (Table 2).^[16–21]^ While the exact mechanism of antitumor immune response which has been thought responsible for this SR is not clear. Severe infection can stimulate excessive production of proinflammatory cytokines, which may alter the immune environment and overcome leukemia-induced immune suppression.^[22]^ COVID-19 typically affects both innate and acquired immune responses. The coronavirus-infected dendritic cells (DCs) showed significant up-regulation of inflammatory chemokines including IFN-ɤ, TNF-α, and IL-6.^[23]^ DCs are the critical antigen-presenting cells (APCs) that initiate the antileukemic immune response by stimulating the differentiation of naïve T cells into the cytotoxic T cells (CTLs).^[24]^ Besides, DCs could stimulate the natural killer and CTLs cells’ activity to eradicate the leukemic cells by increasing the inflammatory chemokines. In this case, we observed significant up-regulation of representative inflammatory chemokines, and speculated abovementioned process might lead to SR in the absence of chemotherapy.

**Table 2:** Summary of SR after COVID-19 infection in patients with hematological malignancies without disease-modifying therapy for each case.

Patient	Age	Gender	Pathology	Disease state	Outcome	Author (year)	Reference
Pt 1	58	Female	Hodgkin lymphoma	New diagnosed	CR	Michał K et al (2022)	^[16]^
Pt 2	61	Male	Hodgkin lymphoma	Refractory	CR	Sarah C et al (2021)	^[17]^
Pt 3	61	Male	Follicular lymphoma	Refractory	CR	Sollini M et al (2021)	^[18]^
Pt 4	57	Female	Acute myeloid leukemia	New diagnosed	CR	Maryam B et al (2022)	^[19]^
Pt 5	63	Female	Acute myeloid leukemia	New diagnosed	CR	Eman Z et al (2021)	^[20]^
Pt 6	28	Male	T-Acute lymphoblastic leukemia	Recurrent	CR	Eman Z et al (2021)	^[20]^
Pt7	67	Male	Chronic lymphocytic leukaemia	New diagnosed	CR	Bülbül H et al (2022)	^[21]^

CR = complete remission, Pt = patient.

In consideration of the high tumor load before the first cycle of chemotherapy in this patient, it seems impossible to achieve CR in a single cycle at reduced dose. This observation suggested that the confirmed COVID-19 infection is one of the possibilities contributed to the CR. We speculated that the COVID-19 infection might impair the interaction between leukemia cells and their micro-environment, or COVID-19 might evoke an antitumor immune process by increasing the production of proinflammatory cytokines, which is also partially directed against the leukemia cells.^[17]^ This could cause an antileukemia effect and even SR in the absence of induction therapy of AML. Another explanation may be that COVID-19 could serve as an oncolytic virus, which is leading to destruction of the leukemia cells causing regression of the disease.^[25]^ This report reveals a possible interplay among COVID-19 infection, inflammation, and tumor biology. However, more extensive investigations are needed to find an explanation for this regression. Additionally, in the absence of disease-modifying therapy, the spontaneous immune related remission is usually transient. Therefore, after SR of AML, continuing treatment by a consolidation regimen or allogeneic transplantation may improve the outcomes and reduce their recurrence risk.

In summary, clinicians should carefully evaluate and discuss the benefits and risks of alternative approaches for each patient in order to provide the best treatment. Larger case series are required to develop reliable treatment guidelines for patients with AML and concomitant COVID-19 infection. This case report suggests that AZA combined with VEN, even at very low doses, is an effective and safe therapy regimen for newly diagnosed AML with severe COVID-19 infection. In addition, we identified the potential effect of COVID-19 evoked immune responses on inducing remission in patients with AML, which may guide the therapy of newly diagnosed AML in patients with severe COVID-19 infection.

Treatment of newly diagnosed AML in patients with concurrent COVID-19 infection can still be a challenge. Azacitidine and venetoclax combination therapy showed superior efficacy in de novo AML patients who are infected with severe COVID-19 unable to withstand intensive chemotherapy. In-depth exploration of the mechanism of antitumor immunity generated by COVID-19 in some patients could guide the treatment selection in de novo AML patients with severe COVID-19 infection.

## Acknowledgments

This work was supported by the Sponsored by Tianjin Health Research Project (Grant No. TJWJ2022XK021), Chinese Society of Clinical Oncology Beijing Xisike Clinical Oncology Research Foundation (Y-SY2021QN-0184 and Y-NCJH202201-0027).

## Author contributions

**Conceptualization:** Yao Qi, Qi Deng.

**Data curation:** Yao Qi, Jing-Yi Li.

**Formal analysis:** Jing-Yi Li, Jia Wang.

**Funding acquisition:** Jia Wang, Qi Deng, Rui Cui.

**Project administration:** Juan Mu, Rui Cui.

**Supervision:** Rui Cui.

**Visualization:** Jia Wang, Juan Mu, Rui Cui.

**Writing – original draft:** Yao Qi.

**Writing – review & editing:** Yao Qi, Jing-Yi Li, Jia Wang, Juan Mu, Qi Deng, Rui Cui.
